# Real-Time Detection of Flu Season Onset: A Novel Approach to Flu Surveillance

**DOI:** 10.3390/ijerph19063681

**Published:** 2022-03-19

**Authors:** Jialiang Liu, Sumihiro Suzuki

**Affiliations:** 1 Department of Epidemiology and Biostatistics, College of Public Health, Temple University, Philadelphia, PA 19122, USA; 2 Department of Preventive Medicine, Rush University Medical Center, Chicago, IL 60612, USA; Sumi-hiro_Suzuki@rush.edu

**Keywords:** public health, surveillance, influenza, change point detection

## Abstract

The current gold standard for detection of flu season onset in the USA is done retrospectively, where flu season is detected after it has already started. We aimed to create a new surveillance strategy capable of detecting flu season onset prior to its starting. We used an established data generation method that combines Google search volume and historical flu activity data to simulate real-time estimates of flu activity. We then applied a method known as change-point detection to the generated data to determine the point in time that identifies the initial uptick in flu activity which indicates the imminent onset of flu season. Our strategy exhibits a high level of accuracy in predicting the onset of flu season at 86%. Additionally, on average, we detected the onset three weeks prior to the official start of flu season. The results provide evidence to support both the feasibility and efficacy of our strategy to improve the current standard of flu surveillance. The improvement may provide valuable support and lead time for public health officials to take appropriate actions to prevent and control the spread of the flu.

## 1. Introduction

Influenza (flu) is an acute viral disease that mainly impacts the respiratory system [1]. The Centers for Disease Control and Prevention (CDC) indicated that approximately 35.5 million people in the United States (USA) were infected with seasonal flu during the 2018–2019 flu season, leading to approximately 34,200 flu-associated deaths and over $10 billion in medical costs, lost productivity, and lost wages [2]. Seasonal flu epidemics are usually caused by type A and type B seasonal influenza viruses [1]. Although seasonal flu epidemics generally occur every year during the fall to winter months, the exact timing of onset is different from season to season. Timeliness in detecting the onset of flu season plays a critical role in flu surveillance to delay the spread and control the impact of the disease. The earlier it is detected, the more time there is to plan and implement proactive prevention strategies against the spread of the disease. For example, with enough lead time, health administrators will be able to make optimal staffing and medical resourcing decisions in preparation for potential surges of patient visits to medical facilities. 

The gold standard for flu surveillance in the USA is the one practiced by the CDC. It uses indicators from laboratories and clinics to determine the onset of flu season, detect the types of flu viruses that are circulating, and measure the impact of flu on hospitalization and deaths. Some of these indicators include the percent of patient visits for influenza-like illness (ILI), percent of respiratory specimens testing positive for flu viruses, rate of influenza-associated hospitalizations, and percent of deaths resulting from pneumonia or influenza [3]. Official flu season onset is determined as the first time the percent of patient visits for influenza-like illness (%ILI) exceeds a predetermined epidemic threshold for two consecutive weeks [4]. An ILI is defined as a body temperature of 100° Fahrenheit or greater and a cough or a sore throat in the absence of a known cause other than influenza [5]. However, due to the time needed to process the data, there is always a delay between the actual time of flu season onset and public dissemination of this information. That is, using the current gold standard of flu surveillance, there is no way to monitor flu activity in real-time, and onset can only be detected after the flu season has already begun. Furthermore, the current flu surveillance system only captures information from patients who are seen by health care providers, and hence, it cannot capture information from patients who have the flu but do not go to healthcare providers. Reasons for not seeking care may vary from being asymptomatic, having a mild infection, or a general reluctance to seek healthcare. Still, it most likely leads to an underestimation of flu activity. Thus, there is a critical need for a surveillance system to monitor flu activity in real- or near real-time fashion to detect the onset of flu season prior to its beginning.

There are two barriers to developing such a surveillance system. First is the availability of accurate real-time data of flu activity. As CDC relies on clinical data from healthcare providers and labs to estimate %ILI, there is always a delay in the availability of CDC %ILI data, sometimes up to a three-week delay [5]. In recent decades, the rapid expansion of the internet has dramatically changed how people search for information, especially about health. Increasingly, health-related information has become available on the internet [6]. The Pew Internet and American Life Project reported that 80% of American internet users indicated that they accessed health-related information using internet search engines (e.g., Google, Yahoo, etc.) [7]. This implies that the frequency of certain online search queries using certain search terms may be used as a proxy for trends and the impact of diseases over time, e.g., seasonal flu activity. A rich body of studies has suggested that internet-based data have great potential to detect epidemic outbreaks such as influenza [6,8,9], Ebola [10], etc. Moreover, since internet-based information can be obtained in real-time, it may serve as a novel, convenient, and cost-effective way to estimate flu activity in a timely manner [6,8,11,12]. For example, a previous study found that a set of Yahoo search queries containing the words ‘flu’ or ‘influenza’ was correlated with virologic and mortality flu surveillance data over several years in the USA [6]. Furthermore, because the internet-based data can capture the patterns of how and when people search flu-related information, it provides more clues and early indications about the shift of the trend of flu activity. The second is a way to determine the early onset of flu season. Several previous studies suggested the feasibility of using a statistical method known as change-point detection to predict the onset of flu season prior to the actual flu beginning [11,13,14]. Although these applications showed great promise, they were not practical because the data sources used were not available in real-time. As a result, any findings were retrospective and only indicated the occurrence of a flu epidemic after the fact. Other flu forecasting models exist that may provide information about impending epidemics, including the duration of the season, the overall burden, and the timing and magnitude of the epidemic peak [15,16,17]. However, these models were not typically designed for early warning or detecting the onset of flu seasons. To this end, we used an established data generation method that combines Google flu-related search volume and past flu activity to simulate real-time estimates of flu activity. We then applied a change point detection method to the generated data to create a flu surveillance system to determine the initial uptick in flu activity indicating the imminent onset of flu season. 

## 2. Materials and Methods

The technical details of our methods are presented elsewhere [18,19]. Briefly, Yang and colleagues created a data generation method called the AutoRegression with General Online (ARGO) model, which combines Google flu-related search query volume data and historical CDC %ILI data to simulate real-time estimates of flu activity (i.e., %ILI) [20]. The weighted version of the historical CDC percentage (%) of ILI data was obtained from CDC’s flu surveillance system (available at www.cdc.gov/grasp/fluview/fluportaldashboard.html, accessed on 1 January 2019. The search volume data of Google flu-related search terms were obtained from Google Correlate (www.google.com/trends/correlate, accessed on 1 January 2019) and Google Trends (www.google.com/trends, accessed on 1 January 2019). Previously, Google launched the Google Flu Trends (GFT) service, which attempted to track flu activity by monitoring and analyzing health care seeking behavior in the form of queries to its online search engine. However, the GFT’s original algorithm for flu activity estimations was found to lack reliability and accuracy compared to those measured by CDC in subsequent years [11,21]. As a result, this service was shut down in August of 2015 [22]. In contrast, the ARGO model exhibits high accuracy in estimating %ILI compared to those reported by CDC with low prediction errors [20]. Therefore, this present study used this method to simulate data from 2007 to 2015. Although it is possible to simulate actual real data, e.g., flu activity today, we used past data to evaluate the accuracy of our methods compared to what actually occurred based on the data from the CDC. We then applied a change point detection method to the ARGO-generated data to detect the onset of flu season. 

**Figure 1:**
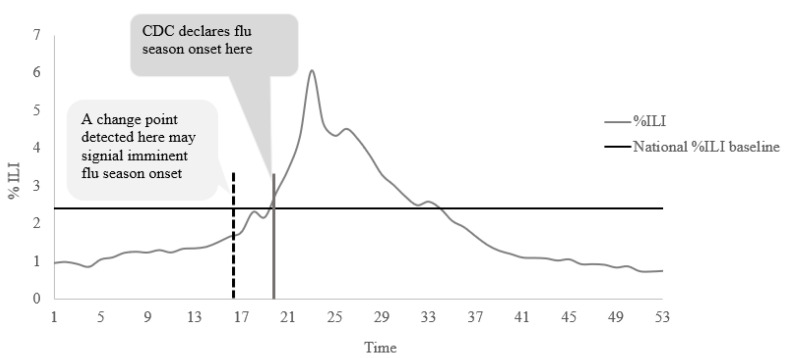
Illustration of using change point detection method for flu season onset detection.

Change point detection is a series of statistical methods used to detect a point in time (called the change point) when the distribution of a time series changes. For this application, the change point indicates a point in time where flu activity from that point is significantly greater than the times prior. Figure 1 depicts the rationale of our detection strategy. We posit that this detected time point (e.g., dotted black vertical line in Figure 1) does not coincide with the CDC-defined flu season onset. Rather, it would occur a few weeks before, when flu activity begins to increase gradually. Thus, by identifying this change point, we would be able to detect the imminent onset of flu season earlier than the actual date of onset. Although many different change point methods exist, we applied a Bayesian online change point detection (BOCPD) method proposed by Adams and MacKay [23]. Change point methods can be classified either as online (real-time) or offline (retrospective), based on when the method is applied to data. Offline methods are applied once all data have been collected, whereas online methods are applied as the data are being collected. Both have their pros and cons. Several attempts have been made to use offline change point detection methods, such as Max-likelihood, Kernel change point analysis model, and Kruskal–Wallis model, to detect the flu outbreak signals from surveillance data [24]. All these methods were found to detect the start of an outbreak effectively. However, all of these are offline methods that use a complete sequence of data to identify the change. Therefore, for the goal of real-time monitoring of flu season onset, the process must be online so that we can detect the onset of flu season in real-time.

The BOCPD method requires that the initial values of parameters of the data distribution are specified a priori. We estimated the initial values of the parameters in late May of each year using historical CDC %ILI data from all past regular flu seasons available up to that year. For example, all historical CDC %ILI data from 1/10/2004 to 5/19/2007 (i.e., week 20 of 2007) were used to estimate the parameters for the detection process during the 2007–2008 season. However, because the H1N1 outbreak in 2009 was not like other flu seasons, the trend of flu activity for this season was very different. Therefore, the CDC %ILI data from 2009 week 21 to 2010 week 20 were excluded in the estimation of parameters for subsequent seasons. In accordance with the Bayesian paradigm, the BOCPD uses posterior probabilities to determine whether a change point occurs at a given time point. We determined that a change point has occurred if the relative change in the maximum a posteriori (MAP, which is the mode of the posterior distribution) between two consecutive time points was greater than or equal to 0.5 [25].

As the BOCPD method was not created specifically for flu surveillance, the process may result in multiple change points. We applied contextual knowledge about the flu and flu seasons to identify the single change point used to identify the onset of flu season. During week 21 to week 39 (spring to summer), any change points detected were considered uninformative because they are almost surely too far from the actual starting date for any conventional flu season. However, if the ARGO %ILI during this period was at or above a predetermined epidemic baseline reported by the CDC for two consecutive weeks, we concluded that flu season had already begun outside the conventional time period of regular flu season (e.g., H1N1 outbreak) and stopped searching for any change points that season. Otherwise, we looked for the change point indicating the onset of the flu season during week 40 to week 20 (fall to winter) of the following year, where conventional flu seasons usually occur. If the first change point detected during this time whose ARGO %ILI was sufficiently close to the epidemic baseline, yet still below the baseline, this point was determined to be the early signal of the imminent flu season onset [19]. Specifically, we computed the relative difference between the ARGO %ILI and the epidemic baseline reported by CDC for a given year to measure if this detected change point is sufficiently close to the epidemic baseline. When the relative difference for a given detected change point during this time frame was less than or equal to 0.4, this change point was identified as the signal of flu season onset. This choice of cutoff reflects that flu activity has significantly increased toward the epidemic baseline. The flu activity level is likely to cross the epidemic baseline to signal the onset of flu season soon.

Based on the criteria above, the detection of an informative change point for signaling an imminent flu season in a given year relies on the cutoff of the relative change in the MAP between two consecutive time points and the cutoff of the relative difference between the ARGO %ILI and the epidemic baseline will lead to a different detection result. To find the optimal combination of these two cutoffs that lead to a high proportion of change points that correctly predicted the imminent onset of flu season, we varied the cutoff of relative change in the MAP between two consecutive time points from 0.1 to 0.8 in increments of 0.1, and the cutoff of the relative difference between the ARGO %ILI and the epidemic baseline from 0.5 to 0.1 in increments of 0.1 and computed the proportion of correct detection for each situation. We found that when the cutoff of the relative change in the MAP between two consecutive time points is equal to 0.5, the cutoff of the relative difference between the ARGO %ILI and the epidemic baseline is equal to 0.4, they produced the optimal detection [19]. Optimality was determined based on the correct number of flu season onsets detected. 

For each study flu season, we used two benchmarks to evaluate the performance of our surveillance system. First, we computed the proportion of change points that correctly predicted the imminent onset of flu season. An informative change point was deemed correct in predicting the flu season onset if it occurred 8 weeks prior to the official flu season onset reported by the CDC. For a given season, if there was no official flu season onset identified by the CDC, an informative change point detected for that season was conservatively deemed incorrect in predicting the onset of flu season. Second, we computed the average number of weeks between the correct change point and the corresponding CDC-reported date of flu season onset among all correctly identified change points. Statistical analysis was performed using R Statistical Software (version 3.4.3) and Matlab (version 9.4).

## 3. Results

**Figure 2:**
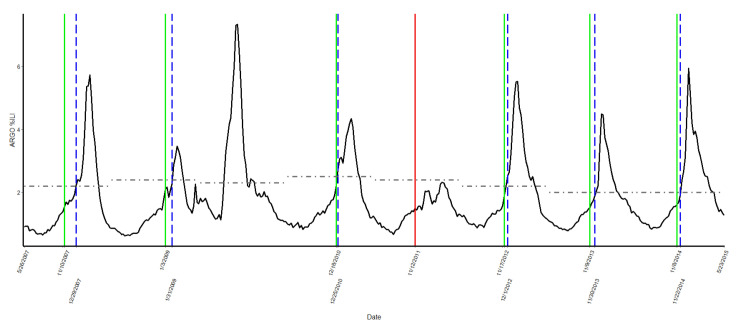
Early signal of flu season onset, 2007–2015 seasons.

Among eight flu seasons considered (2007–2015), we excluded the 2009–2010 season from the analysis as an outlier due to this being the years of the swine flu pandemic [26]. This was the first flu pandemic in the USA in 40 years and should not be considered a “regular” flu season [26]. Figure 2 shows the ARGO estimated %ILI (i.e., black trend line) along with our six correct change points that may signal the imminent flu season onset (i.e., vertical green solid lines) out of seven detected informative change points and the corresponding CDC-reported dates of onsets (i.e., vertical blue dash lines). 

The proportion of correct predictions was high at 86% (6 out of 7 seasons). Additionally, on average, flu season onset was detected three weeks prior to the official CDC-reported date of onset. We incorrectly identified the onset for the 2011–2012 season, but this was a mild flu season where there was no official onset according to the definition used by the CDC. That is, the %ILI never exceeds the epidemic baseline for two consecutive weeks. The specific dates of early detection of flu season onsets are detailed in Table 1.

## 4. Discussion

The present study created a framework for flu surveillance that combines web-based search engine query data with a change point detection method that has the power to predict the onset of an imminent flu season. The novelty of our framework is that we combined two existing methods to overcome two limitations with the gold standard of flu surveillance practiced by the CDC.

First, as the CDC uses data from laboratories, there is always a delay obtaining the ILI for any given week. To this end, the ARGO model is a robust tool to simulate real-time estimates of flu activity based on internet search queries. Using the ARGO model will allow public health officials to obtain the most up-to-date information on flu activity. However, knowing the real-time dynamic of flu activity is not sufficient to provide an early warning of imminent flu season. A second limitation of the current gold standard is that onset of flu season is deemed based on when flu activity levels exceed a predetermined epidemic threshold for two consecutive weeks. As a result, even with real-time flu activity data, there is always a two-week delay in detecting flu season onset. To this end, we utilized the fact that flu activity tends to grow gradually as flu season approaches, and the initial uptick in %ILI data should be an indicator for the eventual onset of flu season. By applying the BOCPD method to the ARGO-generated data, we detected the onset weeks prior. 

The combination of the ARGO model and BOCPD method provided the theoretical framework for this study. However, as no other study has ever used the BOCPD method on flu activity data (prospectively or retrospectively), there was no established convention on applying the method. Thus, our scholarly contribution lies in the granular details necessary for the method to be applied, e.g., choosing the appropriate prior distribution, determining the correct change point that marks the onset of flu season, etc. These details are provided elsewhere [18,19], but the current study proposes a possible solution of expanding the application of BOCPD on infectious disease prevention. The early warning signals detected using our strategy provide an average lead time of three weeks prior to the official onset of flu season. This lead time may allow public health officials to implement targeted strategies for disease prevention in a timely manner. It is said that the time required for planning and implementing a detailed and comprehensive plan for managing seasonal flu takes anywhere from a few days to even weeks [27]. Because the week of flu season onset changes from year to year, it brings uncertainty and a heavy logistic burden to many public health services. Therefore, timely and accurate information tools on monitoring the change of current flu activity play an important role in controlling the spread of the disease. Rapid implementation of flu interventions, such as reducing social and community contacts and increasing home isolation, can significantly curtail the burden of the disease. A previous study found that if flu interventions were implemented within 2 weeks after the introduction of the first infectious case into the community, the peak daily illness attack rate would be reduced by more than 10 fold [28]. Moreover, early detection of flu season onset can improve public awareness of the current risk of flu to increase vaccination rates, which is necessary as flu vaccines are not fully effective until about 2 weeks after the shot [29]. 

However, although our strategy could correctly detect flu season onset on average 3 weeks prior to the official onset, this lead time varied from 1 to 7 weeks across the considered seasons. The BOCPD method relies on the flu activity patterns from prior years to detect the change points for the current year; due to the climate changes, types of seasonal flu virus, the effectiveness of vaccination, prevention intervention, the current flu activity pattern would be very different than the prior years. As a result, the BOCPD method may need more data points coming from the new flu activity pattern to detect this new datum, resulting in a variation in lead time. 

There are several strengths to this study. First, the data sources we used are publicly available on a near real-time basis, and they account for the patients who have the flu but may not go to healthcare providers. As a result, the flu activity data used in the surveillance process tend to be more pragmatic than the flu activity data provided by the CDC. Second, our study shows the feasibility of the BOCPD algorithm to provide early detection of the onset of flu season. The process showed a high level of accuracy and provided a lead time that can be used to implement preventive strategies. Third, the general framework of our strategy can be extended to provide early detection of flu season onset or any seasonal disease at the regional levels in the USA or even in other countries. The seasonal flu has become one of the global health concerns due to the heavy burden it costs. Worldwide, seasonal flu is estimated to result in at least 3 million cases of severe illness and 290,000 respiratory deaths annually [30]. In addition, the effectiveness of the flu surveillance system in many other countries is also being impacted by the same limitations identified in the USA flu surveillance system [31]. Our strategy provides a possible solution that can be adapted to improve flu surveillance globally. 

Despite the many strengths, our new strategy has a few limitations. First, we could only correctly predict the onset in six out of the seven seasons considered. However, the one season where we did not (2011–2012) was notably different from other regular flu seasons. This was a very mild flu season where the %ILI never exceeded the national epidemic baseline for two consecutive weeks. As such, there was no official flu season onset based on the CDC’s criteria. Moreover, we detected the single time point when the %ILI was above the epidemic baseline, which shows the robustness of our method to detect even small changes in flu activity. To be consistent and conservative, we chose to classify this as a failed detection. However, we could easily argue that this is a correct detection. Our goal is to provide a tool for public health officials to take preventive actions to limit the spread of the flu. Our method detected the peak of that year, which would have helped public health officials take action regardless of whether there was an official flu season. Second, our strategy is not designed to estimate the mortality and hospitalization rates related to seasonal flu. Hospitalizations and mortality due to flu are more difficult to capture using search query data because these are events subsequent to infection. As this paper was written in 2022 during the coronavirus disease 2019 (COVID-19) pandemic, we would be remised not to discuss applicability to other infectious diseases. For most other diseases, a direct application of our methods would not be straightforward. The data generation method (ARGO model) relies on the expected seasonality of the disease, and hence, unexpected trends are not captured. However, with COVID-19, real-time data were available every day for most countries, and within the USA, they were available at various levels, including states and cities. Thus, using the ARGO model would not be necessary if there is real-time disease data. Alternatively, if the disease becomes endemic and past data are available, we will be able to apply the ARGO model to determine real-time estimates. The application of the BOPCD method to other data also creates some challenges. We aimed to detect the official start of flu season, defined as two consecutive weeks of flu activity above a predetermined threshold. However, for other diseases, no such threshold exists. Thus, for application to other diseases, we must modify the target for detection. A natural target may be the peak of any surge of the disease. Although it may be less useful than the time of onset, knowing the peak would help to reduce the burden of health professionals and resources available. It may also ease public anxiety toward a disease if the peak of a surge is known. We will determine the best approach to detecting the peak using our methods in our future work. These may be areas of refinement for the broader application of our methods. 

## 5. Conclusions

The results of this study provide evidence to support both the feasibility and efficacy of our strategy to improve the current practice of flu surveillance. We created a novel flu surveillance strategy that allows for the prediction of flu season onset with a valuable lead time for public health officials to take appropriate actions to prevent and control the spread of the flu.

## Figures and Tables

**Table 1:** Dates of the early signal of flu season onset and CDC-reported flu season onset, 2007–2015 seasons.

Seasons	Early Signal of Onset	CDC-Reported Onset	Number of Weeks Prior Official Onset
2007–2008	11/10/2007	12/29/2007	7
2008–2009	01/03/2009	1/31/2009	4
2009–2010	H1N1 outbreak, flu season begun on 8/29/2009		-
2010–2011	12/18/2010	12/25/2010	1
2011–2012	11/12/2011 ^a^	No official onset based on onset definition	-
2012–2013	11/17/2012	12/1/2012	2
2013–2014	11/09/2013	11/30/2013	3
2014–2015	11/08/2014	11/22/2014	2
			Average: 3.17 weeks

^a^ Incorrect detection.

## Data Availability

The data that support the findings of this study are openly available in https://doi.org/10.1073/pnas.1515373112, reference number [20].
